# Risk factors and long‐term postoperative outcomes in patients with postoperative dysphagia after esophagectomy for esophageal cancer

**DOI:** 10.1002/ags3.12566

**Published:** 2022-03-15

**Authors:** Takahito Sugase, Hiroshi Miyata, Keijiro Sugimura, Takashi Kanemura, Tomohira Takeoka, Masaaki Yamamoto, Naoki Shinno, Hisashi Hara, Takeshi Omori, Masahiko Yano

**Affiliations:** ^1^ 53312 Department of Digestive Surgery Osaka International Cancer Institute Osaka Japan

**Keywords:** dysphagia, esophageal cancer, esophagectomy, pneumonia, recurrent laryngeal nerve palsy

## Abstract

**Aim:**

Dysphagia is one of the most common complications after esophagectomy. However, no study has investigated the long‐term postoperative outcomes in patients with postoperative dysphagia. Here, we aimed to identify risk factors for postoperative dysphagia and to investigate long‐term postoperative outcomes in such patients.

**Methods:**

This study included 304 consecutive patients with thoracic esophageal cancer who underwent curative esophagectomy. They were diagnosed with postoperative dysphagia through a contrast videofluoroscopic swallowing study, and postoperative outcomes were compared based on swallowing function.

**Results:**

In total, 112 patients (37%) were diagnosed with postoperative dysphagia. Older age, low BMI, and recurrent laryngeal nerve palsy were identified as independent risk factors for postoperative dysphagia. In the dysphagia group, a significantly larger number of patients developed in‐hospital pneumonia, and hospital stays were also significantly extended. After discharge, 37 (33%) patients with postoperative dysphagia developed pneumonia. Even more than 1 year after esophagectomy, a significantly larger number of patients (24 patients, 21%) with postoperative dysphagia developed pneumonia compared to those without postoperative dysphagia. Postoperative dysphagia was identified as an independent risk factor for out‐of‐hospital pneumonia. Regarding nutritional status, there was no difference in weight loss 1 year after esophagectomy, but significant weight loss was observed 2 years after esophagectomy in the dysphagia group.

**Conclusion:**

Postoperative dysphagia was associated with both preoperative patient factors and surgical factors. Moreover, patients with postoperative dysphagia had long‐term and short‐term pneumonia risk. The personalization of long‐term follow‐up through more aggressive rehabilitation and nutritional guidance is required for patients with postoperative dysphagia.

## Graphical abstract

We aimed to identify risk factors for postoperative dysphagia and to investigate long‐term postoperative outcomes in patients with esophageal cancer. Postoperative dysphagia was associated with both preoperative patient factors and surgical factors and patients with postoperative dysphagia had long‐term and short‐term pneumonia risk.

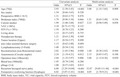

## 1 INTRODUCTION

Radical esophagectomy for thoracic esophageal cancer is still one of the most invasive procedures in the field of gastrointestinal surgery and can cause serious postoperative complications.^1^, ^2^, ^3^, ^4^ The major complications after esophagectomy include anastomotic leakage, pulmonary complications, damage to the recurrent laryngeal nerve, dysphagia, strictures, reflux, and other gastrointestinal symptoms resulting from various risk factors, including both patient and surgical factors.^4^, ^5^, ^6^ Above all, the incidence of postoperative dysphagia has been reported to be relatively high after esophagectomy owing to various factors, such as vocal fold immobility,^7^, ^8^, ^9^ cervical lymph node dissection,^10^ reconstruction route,^11^ and long surgery duration,^12^ which are specific to surgery for esophageal cancer.

When patients develop postoperative swallowing disability, they often struggle to maintain oral nutritional intake, and the majority of them are temporarily dependent on tube feeding or total parenteral nutrition during the early period after esophagectomy.^13^ Furthermore, the presence of postoperative dysphagia has been reported to increase the risk of pneumonia and in‐hospital mortality following esophagectomy.^4^, ^14^ A recent literature review also revealed that patients with postoperative dysphagia might be at an increased risk of in‐hospital pneumonia.^15^ Thus, it might be crucial for patients to undergo an adequate dysphagia assessment and to receive therapeutic interventions in order to achieve better health outcomes. However, in previous studies, postoperative dysphagia had been evaluated in a relatively small number of subjects, and no study has investigated the long‐term postoperative outcomes in patients with dysphagia after esophagectomy.

In the present study, we aimed to investigate the long‐term postoperative outcomes in patients with postoperative dysphagia. Furthermore, we also aimed to identify preoperative patient factors and surgical factors that might be risk factors for postoperative dysphagia.

## 2 PATIENTS AND METHODS

### 2.1 Patients

Between January 2015 and December 2018, 337 consecutive patients with thoracic esophageal cancer underwent esophagectomy at the Osaka International Cancer Institute; among them, 304 who underwent curative esophagectomy were included in this retrospective study. Data on patient characteristics, surgical outcomes, clinicopathological features, and postoperative findings were reviewed from the medical reports. The patients were evaluated using esophagoscopy, computed tomography, or positron emission tomography. The skeletal muscle index (SMI), body fat mass (BFM), and basal metabolic rate (BMR) were measured preoperatively using an InBody 720 Body Composition Analyzer (Biospace, Tokyo, Japan). Difficulty swallowing was evaluated as the chief complaint. The histopathological findings were classified according to the Union for International Cancer Control (UICC) Tumor, Nodes, Metastasis (TNM) classification system.^16^ Recurrent laryngeal nerve palsy was diagnosed when unilateral or bilateral vocal cord paralysis requiring pharmacological treatment with drugs (Clavien‐Dindo classification II ≤). Laryngoscopy performed routinely at the bedside on postoperative days (PODs) 1‐2. The rate of weight loss was assessed relative to the preoperative weight. The study protocol was approved by the Human Ethics Review Committee of Osaka International Cancer Institute (18033‐4).

### 2.2 Radical esophagectomy

The standard procedures for treating thoracic esophageal cancer in this series of patients included transthoracic esophagectomy with upper, middle, and lower mediastinal lymphadenectomy; upper abdominal lymphadenectomy; reconstruction of the gastric tube; and anastomosis of the cervical incision. Cervical lymphadenectomy was not performed in patients with lower thoracic esophageal cancer when an intraoperative histological examination revealed that the recurrent laryngeal nerve lymph nodes were negative. The operator chose to perform video‐assisted thoracic surgery according to the stage of cancer progression. Modified Collard anastomosis via the retrosternal route is the first choice in our institute, but hand‐sewn or circular stapled anastomosis is occasionally performed depending on factors such as short residual esophagus. Gastrostomy or enterostomy with a percutaneous feeding tube is routinely performed, and tube feeding is performed as needed even after discharge.

### 2.3 Swallowing evaluation

A contrast videofluoroscopic swallowing study (VFSS) was routinely performed on PODs 8‐9. In case of the development of postoperative complications, such as pneumonia or anastomotic leakage, this examination was performed after the patient's condition improved. Each patient swallowed 5‐10 ml of iopamidol containing a thickener, followed by 5‐10 ml of iopamidol when significant abnormal findings were absent. The evaluation of liquid aspiration, silent aspiration, residue in the pyriform sinus and vallecula, and epiglottic inversion was performed as a joint effort by gastrointestinal surgeons and speech‐language‐hearing therapists. The degree of aspiration was evaluated based on the amount of thick or thin contrast medium introduced into the trachea. The degree of residuals was also evaluated based on the amount of thick or thin contrast medium remaining in the pyriform sinus or vallecula. When there was a difference in each degree, the evaluation of VFSS was diagnosed with the worse result. Silent aspiration was diagnosed in case of liquid aspiration without the patient choking. Inversion of the epiglottis was evaluated by classifying it into three groups according to the degree (normal, slight, or none).

Patients are firstly classified according to the presence and degree of liquid aspiration, and we then comprehensively evaluate for swallowing function with other findings. Patients with severe aspiration were diagnosed with severe dysphagia. Patients with slight aspiration who had both severe residue and no inversion were diagnosed with severe dysphagia, while those with slight aspiration who didn't have both severe residue and no inversion were diagnosed with mild dysphagia. Patients with no aspiration who had either severe residue or no inversion were diagnosed with mild aspiration, while those with no aspiration who had neither severe residue nor inversion were diagnosed with normal swallowing function (Figure S1). Silent aspiration has not been included in the diagnostic criteria for the comprehensive evaluation. Based on the evaluation of the VFSS results, each patient was scheduled for the postoperative course of oral intake and received swallowing rehabilitation with speech‐language‐hearing therapists.

### 2.4 Statistical analysis

Results are expressed as median (range) for continuous variables and percentage for categorical variables. We retrospectively analyzed the associations between patient data and operative procedures using χ^2^ tests and the Mann‐Whitney *U* test. Cox proportional hazard regression analysis was used for the univariate and multivariate analyses. Each factor with *P* < 0.100 in univariate analysis was analyzed as a variable for the multivariate analysis. All statistical tests were two‐sided, and the threshold for statistical significance was set at *P* = 0.05. Statistical analyses were performed using JMP^®^ Pro 15.1.0 (SAS Institute Inc, Cary, NC, USA).

## 3 RESULTS

### 3.1 Postoperative dysphagia after esophagectomy

The VFSS results of each evaluation for the 304 patients in this study are presented in Table 1. Liquid aspiration was observed in 108 patients (slight; N = 98 [32%], severe; N = 10 [3%]) of which silent aspiration was observed in 85 patients, comprising 76% of the patients with postoperative dysphagia. Residue in the pyriform sinus/vallecula was observed in 136 patients (slight; N = 103 [34%], severe; N = 33 [11%]). The epiglottis was inverted normally in 212 patients (70%), while abnormal inversion was observed in 92 patients (slight; N = 83 [27%], none; N = 9 [3%]).

**TABLE 1:** Evaluation of videofluoroscopic swallowing study

	Total N = 304	Postoperative swallowing function		
		Normal N = 192	Mild dysphagia N = 101	Severe dysphagia N = 11
Liquid aspiration, n (%)				
None	196 (65)	192 (100)	4 (4)	0 (0)
Slight	98 (32)	0 (0)	97 (96)	1 (9)
Severe	10 (3)	0 (0)	0 (0)	10 (91)
Silent aspiration, n (%)	85 (28)	0 (0)	77 (76)	8 (78)
Residue in the pyriform sinus/vallecula, n (%)				
None	168 (55)	145 (76)	21 (21)	2 (18)
Slight	103 (34)	42 (22)	58 (57)	3 (27)
Severe	33 (11)	4 (2)	23 (22)	6 (55)
Inversion of the epiglottis, n (%)				
Normal	212 (70)	167 (86)	43 (43)	2 (18)
Slight	83 (27)	25 (14)	54 (53)	4 (36)
None	9 (3)	0 (0)	4 (4)	5 (46)

**FIGURE 1:**
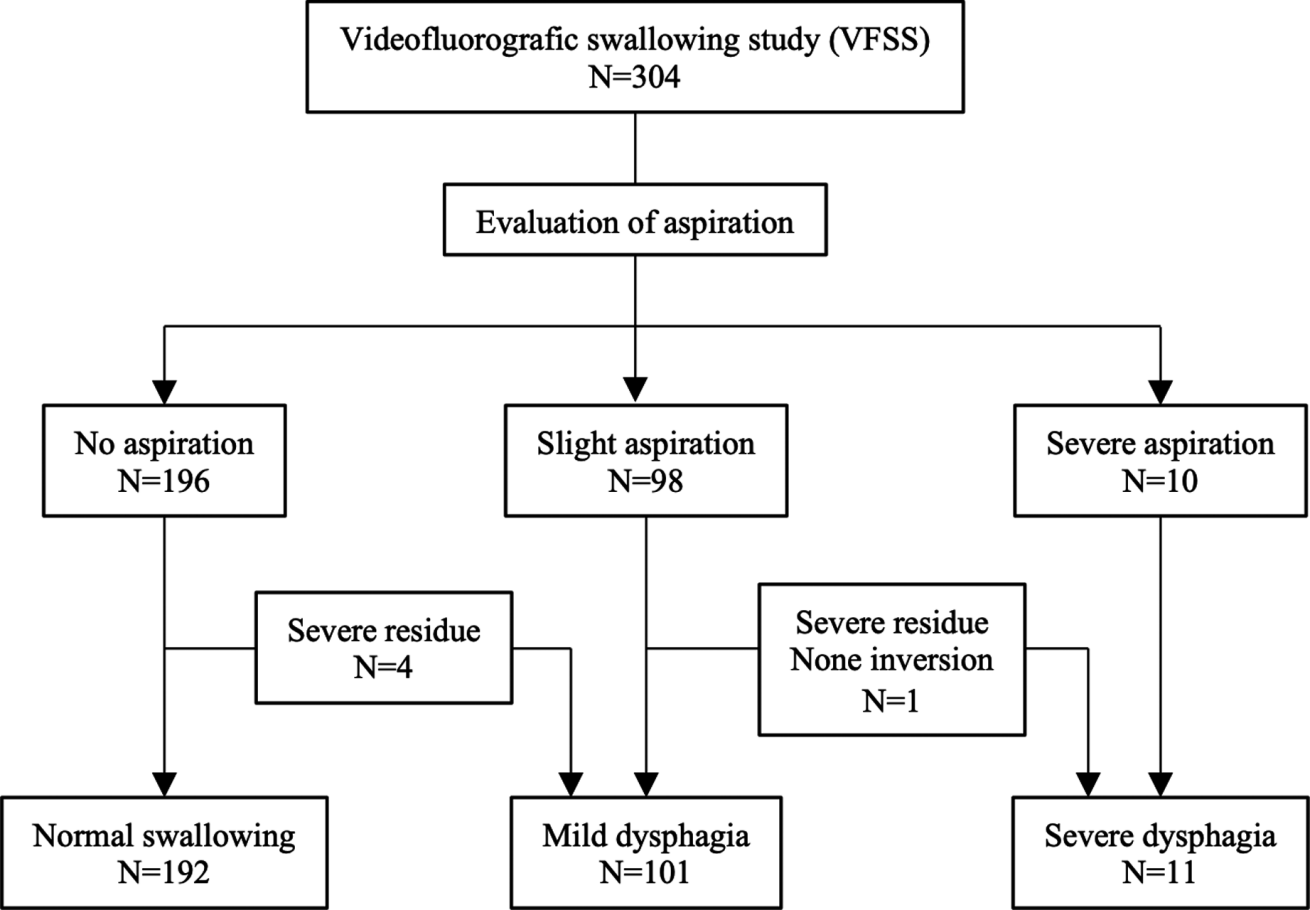
Diagnostic diagram of postoperative dysphagia

Figure 1 shows the diagnostic algorithm for postoperative dysphagia. No liquid aspiration was observed in 196 patients. Four of them were comprehensively diagnosed with mild dysphagia due to severe residue, and others were diagnosed with normal swallowing function. Slight liquid aspiration was observed in 98 patients, of which one patient with slight aspiration was comprehensively diagnosed with severe dysphagia due to severe residue and no inversion. In addition, 10 patients with severe liquid aspiration were also diagnosed with severe dysphagia (Table 1). Finally, normal swallowing function was observed in 192 (63%) patients after esophagectomy (normal group), while postoperative dysphagia was observed in 112 (37%) patients (dysphagia group: mild dysphagia, n = 101; severe dysphagia, n = 11).

### 3.2 Comparison of preoperative patient characteristics, surgical outcomes, and clinicopathological findings

We compared the preoperative patient characteristics between the normal and dysphagia groups (Table 2). Patients with postoperative dysphagia were significantly older (median [range], 66 [41‐80] years vs 70 [42‐81] years; *P* < 0.001), whereas there were no differences in sex and the American Society of Anesthesiologists physical status (ASA‐PS) (*P* = 0.679 and *P* = 0.426, respectively) between groups. The body composition analysis revealed a lower body mass index (BMI) (median [range], 21.4 [15.4‐30.9] kg/m^2^ vs 20.4 [14.1‐34.6] kg/m^2^; *P* = 0.026) and lower SMI (median [range], 9.18 [6.3‐12.8] kg/m^2^ vs 8.69 [6.8‐11.3] kg/m^2^; *P* = 0.040) in the dysphagia group. There were no significant differences in esophageal food obstruction, smoking history, preoperative respiratory function, neoadjuvant therapy, tumor location, histology, and clinical staging between the two groups.

**TABLE 2:** Comparison of preoperative patient characteristics

Total N = 304	Normal N = 192	Dysphagia N = 112	*P*
Age, years, median (range)	66 (41‐80)	70 (42‐81)	<0.001
Sex, n (%)			
Male/Female	154/38 (80/20)	92/20 (82/18)	0.679
ASA‐PS, n (%)			
1/2/3	4/182/6 (2/95/3)	2/103/7 (2/92/6)	0.426
Body composition analysis			
BMI, kg/m^2^, median (range)	21.4 (15.4‐30.9)	20.4 (14.1‐34.6)	0.026
SMI, kg/m^2^, median (range)	9.18 (6.3‐12.8)	8.69 (6.8‐11.3)	0.040
BFM, kg, median (range)	12.4 (3.5‐32.7)	11.1 (1.6‐29.4)	0.154
BMR, kcal, median (range)	1392 (1033‐1814)	1336 (999‐1799)	0.179
Difficulty swallowing, n (%)	70 (36)	49 (44)	0.209
Brinkman index, median (range)	640 (0‐4000)	750 (0‐3000)	0.064
Current smoker, n (%)	74 (39)	53 (47)	0.135
%VC, median (range)	107.9 (67.5‐163.8)	107.4 (65.8‐158.8)	0.192
FEV1%, median (range)	77.7 (53.2‐94.5)	76.5 (40.7‐100)	0.124
Living alone, n (%)	25 (13)	22 (20)	0.123
Neoadjuvant therapy, n (%)			
CT/CRT/None	93/28/71 (48/15/37)	54/21/37 (48/19/33)	0.585
Tumor location, n (%)			
Upper/Middle/Lower	37/101/54 (19/53/28)	24/58/30 (21/52/27)	0.896
Histology			
SCC/non‐SCC	186/6 (97/3)	110/2 (98/2)	0.482
(y)cT, n (%)			
1/2/3/4	82/36/72/2 (43/19/37/1)	35/22/50/5 (31/20/45/4)	0.074
(y)cN, n (%)			
0/1‐2	102/90 (53/47)	47/65 (42/58)	0.060
(y)cM, n (%)			
0/1	174/18 (91/9)	105/7 (94/6)	0.339
(y)cStage, n (%)			
I/II/III/IV	76/40/56/20 (40/21/29/10)	33/26/40/13 (29/23/36/12)	0.354

Abbreviations: ASA‐PS, American Society of Anesthesiologists physical status; BFM, body fat mass; BMI, body mass index; BMR, basal metabolic rate; CRT, chemoradiotherapy; CT, chemotherapy; FEV, forced expiratory volume; SCC, Squamous cell carcinoma; SMI, Skeletal muscle mass index; VC, vital capacity.

Surgical outcomes and clinicopathological findings are presented in Table 3. No significant differences in the surgical procedure or pathological staging were found between the two groups.

**TABLE 3:** Comparison of surgical outcomes and clinicopathological findings

Total N = 304	Normal N = 192	Dysphagia N = 112	*P*
Lymphadenectomy, n (%)			
2‐Field/3‐Field	51/141 (27/73)	32/80 (29/71)	0.705
Reconstruction, n (%)			
Retrosternal/Mediastinal/Antethoracic	153/26/13 (80/13/7)	87/20/5 (78/18/4)	0.463
Anastomosis, n (%)			
Modified Collard/Circular stapler/Hand‐sewn	138/56/1 (72/27/1)	80/30/2 (71/27/2)	0.558
Video‐assisted thoracic surgery, n (%)	98 (51)	46 (41)	0.093
Operation time, minutes, median (range)	512 (329‐751)	524.5 (348‐748)	0.226
Blood loss, ml, median (range)	297 (10‐2635)	287.5 (20‐1335)	0.966
pT, n (%)			
0/1/2/3/4	17/103/17/54/1 (9/53/9/28/1)	12/46/16/38/0 (11/41/14/34/0)	0.212
pN, n (%)			
0/1/2/3	105/54/23/10 (55/28/12/5)	54/39/13/6 (48/35/12/5)	0.656
pM, n (%)			
0/1	176/16 (92/8)	105/7 (94/6)	0.508
pStage, n (%)			
0/I/II/III/IV	13/71/44/39/25 (7/37/23/20/13)	10/28/33/31/10 (9/25/29/28/9)	0.114

### 3.3 Evaluation of risk factors for postoperative dysphagia

The risk of postoperative dysphagia was compared among the potential risk factors. Univariate analyses showed that age (≥70 years), BMI (<18.5), %vital capacity (VC) (<80%), and recurrent laryngeal nerve palsy were significant risk factors for postoperative dysphagia. Multivariable analyses revealed that age (≥70 years), BMI (<18.5), and recurrent laryngeal nerve palsy were independent risk factors for postoperative dysphagia (*P* < 0.001, *P* = 0.043, and *P* = 0.002, respectively) (Table 4).

**TABLE 4:** Univariate and multivariate Cox model analysis for the postoperative dysphagia

	Univariate analysis			Multivariate analysis		
	Odds	95%CI	*P*	Odds	95%CI	*P*
Age (70≦)	3.18	[1.84‐5.50]	<0.001	2.94	[1.75‐4.93]	<0.001
Sex (male)	1.14	[0.62‐2.07]	0.679			
BMI (<18.5)	1.95	[1.09‐3.51]	0.025	1.91	[1.02‐3.59]	0.043
SMI (low*)	1.11	[0.78‐6.76]	0.909			
Esophageal food obstruction	1.36	[0.84‐2.18]	0.210			
Brinkman index (700≦)	1.49	[0.93‐2.38]	0.097	1.33	[0.80‐2.21]	0.268
Current smoker	1.43	[0.89‐2.29]	0.135			
%VC (<80%)	5.38	[1.07‐27.11]	0.042	3.46	[0.64‐18.70]	0.149
FEV1% (<70%)	1.12	[0.62‐2.01]	0.704			
Living alone	1.63	[0.87‐3.06]	0.126			
Neoadjuvant therapy	1.19	[0.73‐1.94]	0.489			
Tumor location (Upper)	1.14	[0.64‐2.03]	0.651			
(y)cStage (3‐4)	1.37	[0.86‐2.19]	0.189			
Non‐video assisted thoracic surgery	1.50	[0.93‐2.40]	0.094	1.30	[0.78‐2.16]	0.308
Lymphadenectomy (3‐Field)	1.11	[0.66‐1.86]	0.705			
Reconstruction (non‐retrosternal route)	1.13	[0.64‐1.99]	0.679			
Anastomosis (Circular stapler)	1.04	[0.62‐1.76]	0.877			
Anastomosis leakage, n (%)	1.27	[0.49‐3.25]	0.624			
Recurrent laryngeal nerve palsy	3.84	[1.91‐7.73]	<0.001	4.48	[1.73‐11.60]	0.002

Abbreviations: BMI; body mass index, FEV, forced expiratory volume; SMI, Skeletal muscle mass index; VC; vital capacity; *male, <7.0 kg/m^2^, female;<5.7 kg/m^2^.

### 3.4 Comparison of short‐term postoperative outcomes

We compared the short‐term postoperative outcomes from the time of surgery until discharge (Table 5). Pneumonia (Clavien‐Dindo classification grade II ≤) was observed in 50 (16%) of 304 patients, and the proportion of these patients was significantly larger in the dysphagia group than the normal group (7% vs 32%, *P* < 0.001). Recurrent laryngeal nerve palsy (Clavien‐Dindo classification grade II ≤) was a significant factor in the dysphagia group (4% vs 15%, *P* < 0.001). Patients with severe dysphagia significantly developed more pneumonia (64%) and recurrent laryngeal nerve palsy (36%) compared to those with mild dysphagia (29% and 13%, respectively). No significant difference in anastomosis leakage was found (6% vs 7%, *P* = 0.623). No postoperative stenosis requiring balloon dilation was observed in each group. The maximum level of C‐reactive protein within POD 7 was significantly higher in the dysphagia than the normal group (13.24 mg/dl vs 15.09 mg/dl, *P* < 0.001). In the dysphagia group, the start of oral intake after esophagectomy was significantly delayed (8 days vs 11 days, *P* < 0.001), and the hospital stay after esophagectomy was significantly longer (17 days vs 21 days, *P* < 0.001), compared with those of the normal group.

**TABLE 5:** Comparison of short‐term and long‐term postoperative outcomes

Total N = 304	Normal N = 192	Dysphagia N = 112	*P* (Normal vs Dysphagia)	Degree of postoperative dysphagia		*P* (Mild vs Severe)
				Mild dysphagia N = 101	Severe dysphagia N = 11	
In‐hospital (short‐term postoperative outcomes)						
Pneumonia, n (%)	14 (7)	36 (32)	<0.001	29 (29)	7 (64)	0.019
Recurrent laryngeal nerve palsy, n (%)	7 (4)	17 (15)	<0.001	13 (13)	4 (36)	0.039
Anastomosis leakage, n (%)	11 (6)	8 (7)	0.623	6 (6)	2 (18)	0.134
Postoperative CRP max, mg/dl, median (range)	13.24 (4.02‐36.93)	15.09 (6.03‐36.49)	<0.001	15.07 (6.03‐36.49)	15.18 (7.92‐30.31)	0.368
Oral intake after surgery, days, median (range)	8 (7‐113)	11 (8‐59)	<0.001	9 (7‐113)	22.5 (14‐46)	<0.001
Postoperative hospital stay, days, median (range)	17 (13‐122)	21 (15‐74)	<0.001	20 (15‐69)	36 (21‐74)	<0.001
Out‐of‐hospital (long‐term postoperative outcomes)						
Pneumonia, n (%)	14 (6)	43 (33)	<0.001	37 (37)	6 (55)	0.246
≦1year	7 (4)	21 (19)	<0.001	17 (17)	4 (36)	0.081
<1 year	9 (5)	24 (21)	<0.001	23 (23)	1 (9)	0.373
Weight change (%, per pre‐op), median (range)						
Post 1‐year	90.5 (65.9‐107.7)	88.3 (71.7‐107.2)	0.228	89.6 (67.4‐108.9)	88.6 (79.5‐96.5)	0.893
Post 2‐year	90.8 (58.6‐112.5)	87.5 (69.8‐105)	0.039	88.7 (66.7‐108.2)	87.9 (71.8‐97.7)	0.705
Death from pneumonia, n (%)	0 (0)	6 (5)	0.002	6 (6)	0 (0)	0.530

Abbreviation: CRP, C‐reactive protein.

### 3.5 Comparison of long‐term postoperative outcomes

We also compared the short‐term postoperative outcomes after discharge (Table 5). In this study, the median follow‐up period was 37.0 (range 1‐76) months after discharge. The development of pneumonia in the patients with recurrence was analyzed until the date of recurrence to exclude the effects of disease‐related pneumonia. Out‐of‐hospital pneumonia (Clavien‐Dindo classification grade II ≤) was observed in 48 patients, and the number of such patients was significantly larger in the dysphagia group (*P* < 0.001) than the normal one. Between the groups, a significantly larger number of patients in the dysphagia group developed pneumonia within 1 year after esophagectomy (*P* < 0.001). A significant number of patients also developed pneumonia in the dysphagia group (*P* < 0.001) even after more than 1 year following esophagectomy. Regarding nutrition, no difference in weight loss was observed 1 year after esophagectomy (90.5% vs 88.3%, *P* = 0.228), while significant weight loss was noted in the dysphagia group 2 years after esophagectomy (90.8% vs 87.5%, *P* = 0.039), when compared with the normal group. Patients with severe dysphagia tended to develop more pneumonia (≦1 year) (55%) compared to those with mild dysphagia (37%), but there were no differences in body weight change. Death from pneumonia was observed in six patients, all of whom had postoperative dysphagia; median survival was 19 months (range 9‐36 months), while no relationship between postoperative pneumonia and long‐term survival was observed (Figure S2).

### 3.6 Evaluation of risk factors for postoperative pneumonia

Potential risk factors were compared for the risk of in‐hospital and out‐of‐hospital pneumonia. Univariate analyses revealed that age (≥70 years), current smoking, non‐retrosternal route, anastomosis with a circular stapler, long operation time (≥10 hours), recurrent laryngeal nerve palsy, and postoperative dysphagia were significant risk factors for in‐hospital pneumonia. In the multivariable analyses, age (≥70 years), long operation time (≥10 hours), recurrent laryngeal nerve palsy, and postoperative dysphagia were identified as independent risk factors for in‐hospital pneumonia (Table 6).

**TABLE 6:** Univariate and multivariate Cox model analysis for in‐hospital pneumonia

	Univariate analysis			Multivariate analysis		
	Odds	95%CI	*P*	Odds	95%CI	*P*
Age (≦70)	2.93	[1.58‐5.43]	<0.001	3.08	[1.35‐7.05]	0.008
Sex (male)	1.54	[0.66‐3.63]	0.320			
Preoperative BMI (<18.5)	1.31	[0.62‐2.75]	0.476			
Brinkman index (≦700)	1.79	[0.96‐3.34]	0.066	1.51	[0.65‐3.49]	0.336
Current smoker	1.99	[1.08‐3.68]	0.027	2.23	[0.98‐5.09]	0.058
%VC (<80%)	3.18	[0.73‐13.75]	0.122			
FEV1% (<70%)	1.59	[0.78‐3.23]	0.200			
Living alone	1.13	[0.37‐2.07]	0.755			
Neoadjuvant therapy	1.21	[0.63‐2.30]	0.569			
Tumor location (Upper)	1.17	[0.54‐2.57]	0.690			
non‐video assisted thoracic surgery	1.13	[0.62‐2.08]	0.684			
Lymphadenectomy (3‐Field)	1.08	[0.54‐2.16]	0.821			
Reconstruction (non‐retrosternal route)	2.02	[1.03‐3.96]	0.040	1.04	[0.38‐2.83]	0.944
Anastomosis (Circular stapler)	2.24	[1.19‐4.21]	0.012	2.15	[0.85‐5.47]	0.108
Operation time (≦10 h)	2.84	[1.35‐5.97]	0.006	3.06	[1.12‐8.36]	0.030
Blood loss (≦500 ml)	1.45	[0.74‐2.84]	0.280			
pStage (3‐4)	1.08	[0.57‐2.03]	0.812			
Recurrent laryngeal nerve palsy	6.80	[3.29‐14.04]	<0.001	32.87	[9.69‐111.44]	<0.001
Postoperative swallowing function (Dysphagia)	6.02	[3.07‐11.81]	<0.001	4.05	[1.78‐9.21]	<0.001

Abbreviations: BMI, body mass index; FEV, forced expiratory volume; VC, vital capacity.

Univariate analyses revealed that age (≥70 years), the Brinkman index (≤700), recurrent laryngeal nerve palsy, and postoperative dysphagia were significant risk factors for out‐of‐hospital pneumonia. In the multivariable analyses, postoperative dysphagia was identified as an independent risk factor for out‐of‐hospital pneumonia (Table 7).

**TABLE 7:** Univariate and multivariate Cox model analysis for out‐of‐hospital pneumonia

	Univariate analysis			Multivariate analysis		
	Odds	95%CI	*P*	Odds	95%CI	*P*
Age (≦70)	2.39	[1.28‐4.47]	0.006	1.44	[0.72‐2.86]	0.298
Sex (male)	2.25	[0.85‐5.95]	0.104			
Preoperative BMI (<18.5)	1.39	[0.66‐2.94]	0.383			
Brinkman index (≦700)	2.03	[1.07‐3.86]	0.030	1.79	[0.90‐3.56]	0.099
Preoperative current smoker	1.49	[0.80‐2.76]	0.210			
%VC (<80%)	1.32	[0.16‐10.99]	0.797			
FEV1% (<70%)	1.70	[0.83‐3.46]	0.146			
Living alone	1.32	[0.59‐2.95]	0.493			
Neoadjuvant therapy	1.11	[0.58‐2.09]	0.756			
Tumor location (Upper)	1.06	[0.49‐2.26]	0.885			
non‐video assisted thoracic surgery	1.38	[0.74‐2.57]	0.305			
Reconstruction (retrosternal route)	1.02	[0.48‐2.17]	0.968			
Anastomosis (Circular stapler)	1.26	[0.64‐2.46]	0.504			
pStage (3‐4)	1.17	[0.62‐2.21]	0.639			
Recurrent laryngeal nerve palsy	3.00	[1.20‐7.47]	0.018	1.75	[0.65‐4.73]	0.271
Postoperative swallowing function (dysphagia)	8.12	[3.93‐16.76]	<0.001	6.76	[3.18‐14.37]	<0.001

Abbreviations: BMI, body mass index; FEV, forced expiratory volume; VC, vital capacity.

## 4 DISCUSSION

Postoperative dysphagia is one of the most common complications after esophagectomy.^4^, ^17^ However, most of the studies related to dysphagia after esophagectomy included a small number of subjects.^15^ This study included 304 consecutive patients who underwent radical esophagectomy for esophageal cancer in our institute, among whom 112 (37%) patients were diagnosed with postoperative dysphagia. Among preoperative patient factors and surgical factors, age (≥70 years), BMI (<18.5), and recurrent laryngeal nerve palsy were identified as independent risk factors for postoperative dysphagia. Furthermore, a larger number of patients with postoperative dysphagia developed pneumonia and were at a severe nutritional risk even in the long term after esophagectomy. This study is the first to report on health‐related long‐term postoperative outcomes in patients with postoperative dysphagia after esophagectomy.

Previous studies have investigated the risk factors for postoperative dysphagia diagnosed by a VFSS.^10^, ^11^, ^12^, ^13^, ^18^ Most of them reported on surgical risk factors for postoperative dysphagia. Some studies that included less than 30 patients with esophageal cancer showed that postoperative dysphagia was associated with three‐field lymph node dissection^10^, ^13^ or retrosternal reconstruction.^11^ Lee et al also showed that operation time greater than or equal to 6 hours and vocal cord paralysis were risk factors for subglottic aspiration in 118 patients who underwent esophagectomy for esophageal cancer.^12^ Regarding patient factors, Mann et al showed that swallowing dysfunction after esophagectomy was correlated with older age in 129 patients with esophageal cancer.^18^ All the previous studies identified risk factors from among either patient factors or surgical factors in a relatively small number of subjects. In the present study, we identified risk factors for postoperative dysphagia among preoperative patient factors and surgical factors in the largest number of subjects. Among the surgical factors, recurrent laryngeal nerve palsy was demonstrated to be an independent risk factor for postoperative dysphagia, while the surgical procedure was not associated with postoperative dysphagia. Since recurrent laryngeal nerve palsy manifests as hoarseness, aphonia, or even severe respiratory problems,^19^ nerve injury should be avoided during esophagectomy in order to reduce the incidence of this condition.^20^ However, postoperative dysphagia after esophagectomy is often observed even if postoperative complications, including recurrent laryngeal nerve palsy, do not occur. In this study, older age and low BMI were identified as independent risk factors for postoperative dysphagia. These results suggest that postoperative dysphagia can occur depending on the preoperative condition of the patient regardless of surgical factors. Therefore, preoperative patient factors and surgery‐related factors can be responsible for postoperative dysphagia.

Postoperative pulmonary complications, such as pneumonia and acute respiratory distress syndrome, are frequent complications following esophagectomy, occurring in approximately 17% to 67% of all patients.^21^, ^22^, ^23^ Advanced age has been considered a significant risk factor for postoperative complications after esophagectomy. Moreover, the presence of dysphagia has been determined to be associated with health‐related outcomes during hospitalization following esophagectomy.^4^ Lee et al^12^ observed an increased risk of pneumonia in patients who aspirated. In the present study, age (≥70 years), operation time (≥10 hours), recurrent laryngeal nerve palsy, and postoperative dysphagia were identified as independent risk factors for in‐hospital pneumonia in this population. Current smoking also tended to be a risk factor for in‐hospital pneumonia. During the early postoperative period, pneumonia may develop due to the combined effects of patient and surgical factors. However, while studies have investigated the postoperative outcomes in patients with postoperative dysphagia during the early period after esophagectomy, no study has investigated the long‐term outcomes.

The development of long‐term pneumonia after esophagectomy in patients with postoperative dysphagia has not been fully investigated. The present study showed that 33% of patients with postoperative dysphagia developed pneumonia during the outpatient follow‐up period. Even more than 1 year after esophagectomy, a significantly larger proportion of patients (21%) with postoperative dysphagia developed pneumonia compared with those without postoperative dysphagia. Moreover, nine of 11 patients with severe dysphagia developed postoperative pneumonia. The multivariate analysis also showed that postoperative dysphagia was an independent factor for outpatient pneumonia. No association with preoperative patient factors or surgical factors was found, unlike in the case of short‐term postoperative outcomes. Thus, we found that postoperative dysphagia, diagnosed early after esophagectomy, could be associated with long‐term pneumonia. The condition of patients with recurrent laryngeal nerve palsy, related to early postoperative pneumonia, usually improves within the first few months after surgery; however, these results might support the possibility that some patients with postoperative dysphagia have prolonged swallowing dysfunction for a long period after esophagectomy.

Regarding nutrition after esophagectomy, clinically significant weight loss has been considered a common problem.^24^, ^25^, ^26^ However, few studies have reported an association between postoperative dysphagia and long‐term weight loss after esophagectomy. The present study showed that some patients in the dysphagia group experienced significant weight loss 2 years after esophagectomy. These results suggest that dysphagia and long‐term postoperative weight loss are closely related. Although no validated data related to the symptoms associated with dysphagia were investigated in the present study, postoperative dysphagia diagnosed early after esophagectomy might have caused weight loss due to the difficulty in oral intake even 2 years after surgery.

Our study has several limitations. First, this was a retrospective study conducted at only one institution. However, compared with previous studies, this study included a large number of patients with esophageal cancer who underwent curative esophagectomy. Second, although almost all patients underwent a VFSS after esophagectomy, few patients underwent a VFSS before esophagectomy or in the long term after esophagectomy. Therefore, we could not directly compare the change in swallowing function before and after esophagectomy and monitor long‐term changes in patients with postoperative dysphagia. This study showed that postoperative dysphagia was associated with both preoperative patient factors and surgical factors. Therefore, patients with postoperative dysphagia caused by preoperative patient factors may have already had swallowing dysfunction before esophagectomy. There is a need for screening and preoperative interventions, in addition to the improvement of surgical procedures, in patients with preoperative dysphagia.

In conclusion, the present study showed that postoperative dysphagia was independently associated with preoperative patient factors, including older age and low BMI, and surgical factors, including recurrent laryngeal nerve palsy. Moreover, patients with postoperative dysphagia were at a risk of pneumonia and weight loss as long‐term postoperative outcomes. These results might provide helpful evidence that can aid in personalizing the long‐term follow‐up of patients with dysphagia through more aggressive rehabilitation and nutritional guidance.

## DISCLOSURE

Conflict of Interest: The authors declare no conflict of interests for this article.

Ethical Statements: The protocol for this research project has been approved by a suitably constituted Ethics Committee of the institution, and it conforms to the provisions of the Declaration of Helsinki. The Human Ethics Review Committee of the Osaka International Cancer Institute, Approval No. 18033‐4.

## Supporting information

Figure S1

Figure S2
