# Supplementary figures and images for: Risk factors and long‐term postoperative outcomes in patients with postoperative dysphagia after esophagectomy for esophageal cancer
Source: Ann Gastroenterol Surg. 2022 Mar 15;6(5):633–42. doi: 10.1002/ags3.12566 (PMC9444858; doi:10.1002/ags3.12566)

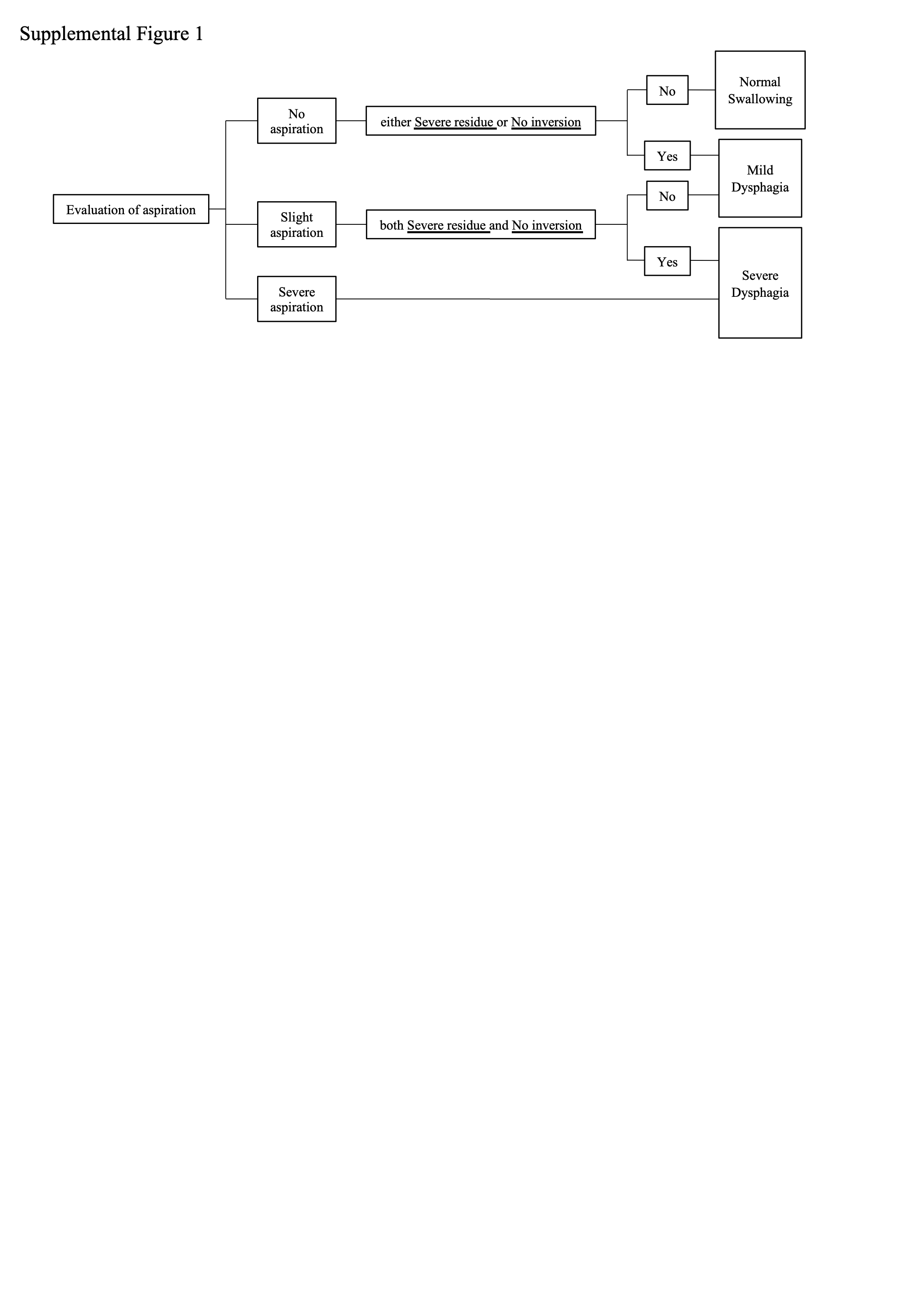

Supplement: Supplementary file 1 — Figure S1 [file AGS3-6-633-s001.tiff]

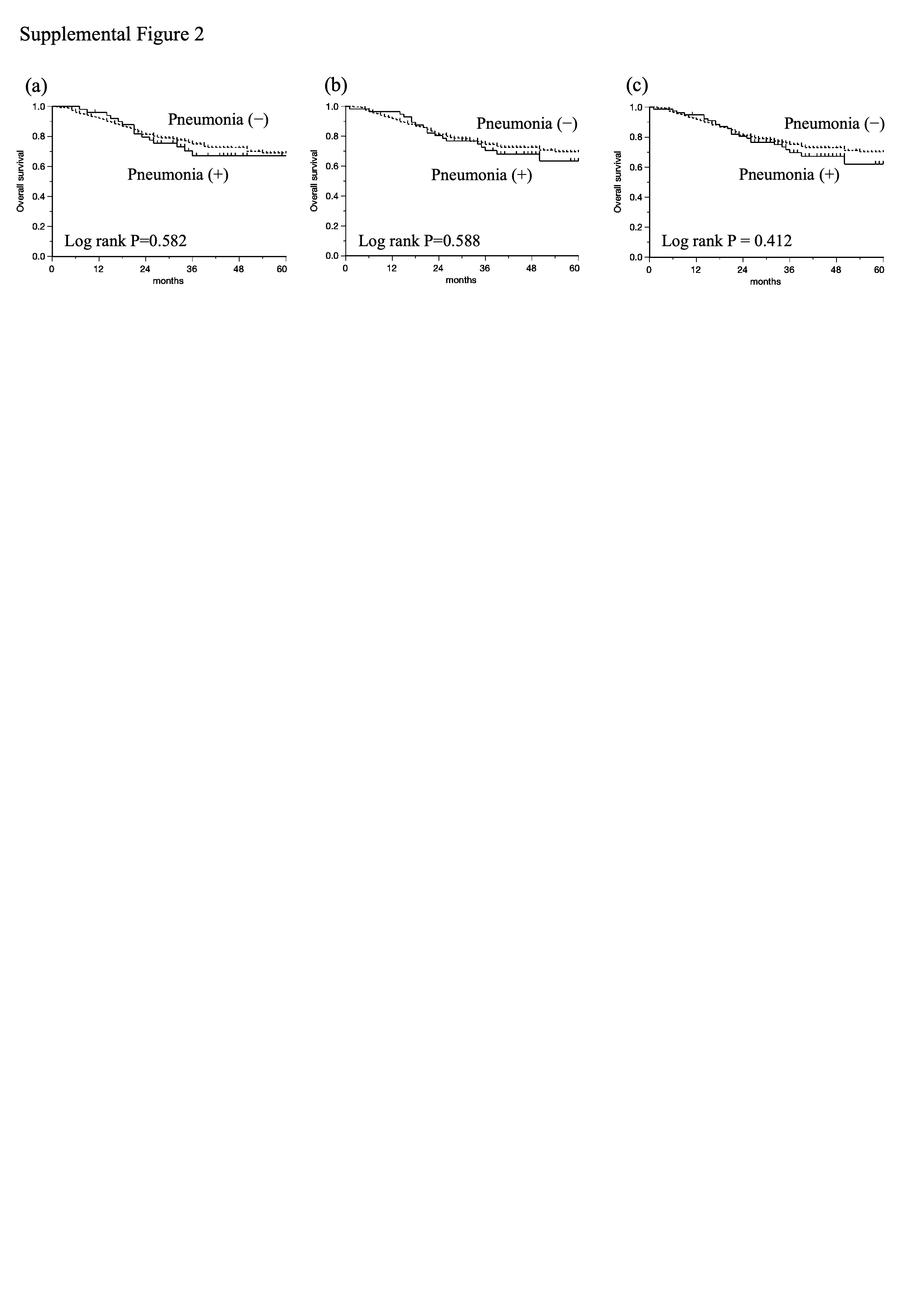

Supplement: Supplementary file 2 — Figure S2 [file AGS3-6-633-s002.tiff]
